# *NlATG1* Gene Participates in Regulating Autophagy and Fission of Mitochondria in the Brown Planthopper, *Nilaparvata lugens*

**DOI:** 10.3389/fphys.2019.01622

**Published:** 2020-01-31

**Authors:** Feifei Yu, Peiying Hao, Chenglong Ye, Yalin Feng, Kun Pang, Xiaoping Yu

**Affiliations:** Zhejiang Provincial Key Laboratory of Biometrology and Inspection & Quarantine, College of Life Sciences, China Jiliang University, Hangzhou, China

**Keywords:** autophagy, autophagy-related gene, mitochondrion, mitophagy, *Nilaparvata lugens*, pest control, RNA interference, survival

## Abstract

Autophagy plays multiple roles in regulating various physiological processes in cells. However, we currently lack a systematic analysis of autophagy and the autophagy-related gene 1 *ATG1* in the brown planthopper (BPH, *Nilaparvata lugens*), one of the most destructive of the insect pests of rice. In this study, the full-length cDNA of an autophagy-related gene, *NlATG1*, was cloned from BPH. Real-time qPCR (RT-qPCR) revealed that this *NlATG1* gene was expressed differently across developmental stages, at higher levels in nymphs but lower levels in adults. RNA interference with dsNlATG1 significantly decreased the mRNA level of the target gene to 14.6% at day 4 compared with that of the dsGFP control group. The survival of the dsNlATG1-treated group decreased significantly from day 4 onward, dropping to 48.3% on day 8. Examination using transmission electron microscopy (TEM) showed that epithelial cells of the BPH’s midgut in the dsNlATG1-treated group had less autophagic vacuoles than did the dsGFP control, and knockdown of *NlATG1* clearly inhibited the starvation-induced autophagy response in this insect. RNA interference of *NlATG1* upregulated the *NlFis1* gene involved in mitochondrial fission, leading to reductions in mitochondrial width and area. Furthermore, knockdown of *NlATG1* also decreased the ATP content and accumulation of glycogen. Together, these results demonstrate that the *NlATG1* gene participates in regulating autophagy and fission of mitochondria in the brown planthopper, making it a potentially promising target for pest control given its key role in autophagy, including maintaining the normal structure and function of mitochondria.

## Introduction

Autophagy is a process of bulk or selective degradation that depends on lysosomes in cells (Van der Vaart et al., 2008). Autophagy plays a pivotal role in maintaining physiological homeostasis and the survival of cells by removing soluble macromolecules from the cytoplasm, organelles, and microorganisms in the cell (Van der Vaart et al., 2008; Jing and Lim, 2012; Zhan et al., 2018). Autophagy may be induced through chemical pesticides, nutrient deficiencies (starvation), hormone stimulation, and ionizing radiation, among other factors (Mcphee and Baehrecke, 2009; Wong et al., 2013). Furthermore, both selective and non-selective autophagy exist. The selective pathway targets organelles that are surplus or damaged, such as the mitochondria, endoplasmic reticulum, and ribosome. The non-selective pathway is always induced by starvation, ensuring the normal operation of cells (Papinski and Kraft, 2016). Autophagy-related genes (ATGs) and proteins are key executive factors in both types of autophagy, having been first found in *Saccharomyces cerevisiae* (Liu et al., 2005). Since then, at least 30 ATGs have been identified (Mari and Reggiori, 2016).

In insects, autophagy occurs in many physiological and morphological processes, including the remodeling of organs (Franzetti et al., 2012; Khoa and Takeda, 2012; Liu et al., 2013), molting (Franzetti et al., 2016), responding to starvation (Liu et al., 2007; Wu et al., 2011), and inhibiting the infection of pathogens (Cherry, 2009; Yue et al., 2015; Wang et al., 2016; Santana et al., 2019). *ATG1* is one of the known ATGs, being essential for the initiation of autophagy, by forming an important complex with other proteins. This complex promotes the initiation of autophagy, integrating and transmitting signals from upstream to downstream of the autophagy pathway (Löffler et al., 2011; Shang and Wang, 2011; Wang and Kundu, 2017; Chun and Kim, 2018).

In *Bombyx mori*, *Drosophila melanogaster*, and *Spodoptera litura*, ATG1 interacts with other autophagy-related proteins and enhances autophagy degradation, and RNA interference of the *ATG1* gene leads to a decrease in autophagy levels (Scott et al., 2007; Hou et al., 2008; Casati et al., 2012; Zhang et al., 2016). Yet, according to some studies, mechanisms inducing autophagy could operate differently in different species or under different situations. For example, *ATG1* was identified as an essential gene for the onset of autophagy in both yeast and Drosophila (Chew et al., 2015; Ma et al., 2018), whereas it was also found that, in mice, autophagy could be stimulated via a process that does not require the involvement of ATG1/ULK1 (Wong et al., 2013). Similarly, although the importance of ATG1 and its complex has been confirmed in Arabidopsis during this plant’s autophagy (Suttangkakul et al., 2011; Li et al., 2014), recently, they were found not to be necessary for activating autophagy under prolonged fixed-carbon stress (Huang et al., 2019). By analyzing the genomes of 40 non-unikonts, Földvári-Nagy et al. (2014) found that some of these genomes do not encode typical ATG1 proteins and that the members of the complex are highly variable, thus indicating that some species possess different autophagy-inducing mechanisms. In sum, the regulation of autophagy is a complex phenomenon, and the role of ATG1 in the mechanism of one species cannot be easily generalized to others. Among the insects, autophagy, including the *ATG1* gene, has been well studied, albeit that the focus has been mainly on model systems, such as Drosophila and silkworm. Both of these are completely metamorphosed insects, whose organs, such as mouthparts and midgut, undergo remolding during phases of metamorphosis. Since no external nutrition is relied upon during the pupal stage, autophagy is thought to be necessary to remobilize nutrients stored in the pupa. By contrast, the brown planthopper (BPH, *Nilaparvata lugens*) is an insect that is incompletely metamorphosed and that does not pass through a pupal stage in its life cycle, so its piercing-sucking mouthparts and its mode of feeding are unchanged for its entire lifetime. Further, the BPH only feeds on rice plants, mainly sucking their phloem sap with its piercing–sucking mouthparts; hence, food ingested by the BPH differs considerably from that obtained by insects with chewing or scratching mouthparts. Therefore, in terms of modes of feeding behavior and metamorphosis, the BPH clearly differs from the well-studied Drosophila and silkworm. Additionally, BPH is one of the most destructive insect pests of rice crops (Park et al., 2007; Wang et al., 2008; Liu et al., 2015; Xue et al., 2018), but the functioning of autophagy and its related genes in this insect remain, surprisingly, unknown.

In this study, we cloned the full-length cDNA of the autophagy-related gene *NlATG1* and analyzed its expression pattern using real-time qPCR (RT- qPCR). We also explored the function of *NlATG1* by knocking down its expression via the RNA interference (RNAi) technique. Our results showed that applying RNAi to *NlATG1* inhibited autophagy and glycogen metabolism and decreased the ATP content and survival of BPH. Therefore, the *NlATG1* gene is required for autophagy and is a promising target for developing a novel BPH control strategy.

## Materials and Methods

### Insect Organisms

The BPH insects used in this study were maintained for approximately 100 generations on the susceptible rice variety TN1. All these BPH insects were reared under the same conditions, at 26 ± 2°C, with humidity of 80 ± 5% and under a 12-h/12-h light/dark photoperiod.

### RNA Extraction and First-Strand cDNA Synthesis

Nymphs (1st to 5th instar) and adults (1–9 days after eclosion) were collected and immediately frozen in liquid nitrogen, using 10 adults or 30–50 nymphs per replicate. Total RNA was extracted using the MiniBEST Universal RNA Extraction Kit (TAKARA, Tokyo, Japan), according to the manufacturer’s instructions. The RNA was then quantified with a Nanodrop 2000 system (Thermo, Wilmington, DE, United States) and its integrity and purity determined by electrophoresis in a 1% agarose gel. The first strand of cDNA was reverse transcribed, using 1 μg of total RNA as the template, with the PrimeScript RT reagent Kit with gDNA Eraser (TAKARA, Tokyo, Japan), which removes genomic DNA in 2 min at 42°C before cDNA synthesis.

### Cloning of the Full-Length cDNA of the *ATG1* Gene

According to the transcriptome sequence we obtained, part of the *ATG1* gene’s core sequence in BPH was selected and identified on the NCBI website via sequence alignment. The PCR primers NlATG1-F and NlATG1-R (Supplementary Table S1) were designed using Primer Premier 5.0 software, and the synthesized first-strand cDNA served as the template for PCR amplifications. This amplification reaction system consisted of 50 μL (25 μL of PCR Mix, 2 μL each of 10 μmol/L positive and negative primers, 2 μL of cDNA template, and 19 μL of ddH_2_O). The PCR reaction procedure had the following conditions: 94°C for 4 min, 30 amplification cycles (94°C for 30 s, 55°C for 30 s, and 72°C for 3 min), and 72°C for 10 min. Each PCR amplification product was separated by 1% agarose gel electrophoresis, and the DNA fragment of interest was recovered with the MiniBEST Agarose Gel DNA Extraction Kit (TAKARA, Tokyo, Japan), cloned into the pMD18-T vector, and then transformed into competent cells of *Escherichia coli* JM109. The positive clones were selected and sent to the Sunny Biotechnology Company (Shanghai, China) for sequencing. Sequencing results were verified by running alignments with the original sequences, performed in DNAMAN software.

To clone the full-length cDNA of the *ATG1* gene, 5′-RACE (rapid amplification cDNA ends) and 3′-RACE was carried out using the BD SMART^TM^ RACE cDNA Amplification Kit (Clontech, Palo Alto, CA, United States). The outer primers (i.e., NlATG1-5′RACE outer/NlATG1-3′RACE outer) and the internal primers (i.e., NlATG1-5′RACE inner/NlATG1-3′RACE inner) were designed separately (Supplementary Table S1). According to the RACE kit instructions, the 3′ and 5′ ends of the target gene were amplified via nested PCR, and the ensuing product was separated by agarose gel electrophoresis. The DNA fragment of interest was recovered, cloned into the pMD18-T vector, and transformed into competent cells of *E. coli* JM109. The positive clones were selected and sent to Sunny Company for sequencing. These sequencing results were then spliced using DNAMAN software to obtain the full-length cDNA sequence of *ATG1* (*NlATG1*). The full-length cDNA of *NlATG1* was verified by PCR, with the primers NlATG1-FL-F and NlATG1-FL-R (Supplementary Table S1) designed for the two ends of the spliced sequence, respectively.

### Sequence Analysis of *NlATG1*

After the full-length cDNA of *NlATG1* was cloned, its open reading frame (ORF) was found using the ORF finder tool^1^. Next, an NCBI BLASTX search was performed for the amino acid sequence homology alignment. The molecular weight and theoretical isoelectric point of a protein were analyzed by ExPASy^2^, and its signal peptide was predicted using the online SignalP 4.1 server^3^. All protein domains were predicted in the PROSITE database^4^. A phylogenic tree of ATG1 was constructed based on the amino acid sequences from the BPH and other species by using the neighboring-joint (NJ) method in MEGA 5.0. Testing of this phylogeny was done by bootstrapping (*n* = 1000 replications), for which percentage values larger than 50 were shown on each node of the tree. Multiple sequence alignments were performed in DNAMAN software.

### Real-Time-qPCR Analysis of Gene Expression

To analyze *NlATG1* gene expression in BPH during its developmental stages, nymphs (1st, 2nd, 3rd, 4th, and 5th instar) and adults (females and males at 1, 3, 5, 7, and 9 days post-eclosion) were sampled separately, with 10 adults or 30–50 nymphs used per replicate. To analyze *NlATG1* gene expression in different parts or tissues, 30 individuals of 5th instar nymphs were sampled and dissected (head, thorax, midgut, ovary, and fat body).

RNA extraction and reverse transcription of the first strand of cDNA was carried out as described in subsection 2.3 above. The genes’ specific primers for the RT-qPCR can be found in Supplementary Table S1, and the *RPS11* gene of BPH served as an internal reference to detect the relative expression level of the target genes in each sample (Yuan et al., 2014). Three independent biological replicates and three technical replicates were set up in the experiment. The relative expression level of a given gene was calculated using the 2^–Δ^ ^Δ^ ^Ct^ method (Livak and Schmittgen, 2001).

### Double-Stranded RNA Synthesis

Primers of dsNlATG1-F and dsNlATG1-R (Supplementary Table S1) for the synthesis of double-stranded RNA interference fragments were designed based on the full-length cDNA sequence (i.e., from 272 to 993, 722 bp). A protective base (GGATCC) and a T7 promoter (TAATACGACTCACTATA) were added to the 5′ end of the specific primer and cloned into pMD18-T vector, which was then transferred into JM109 for sequencing. In order not to affect the transcription level after RNAi, the interference fragment did not include the fragment for RT-qPCR. The dsRNA was synthesized by following the instructions of the MEGAscript^®^ T7 High Yield Transcription Kit (Ambion, Austin, TX, United States). To purify the dsRNA, 30 μL of ddH_2_O and 30 μL of the LiCl Precipitation Solution were added to the dsRNA reaction system. This was allowed to sit at –20°C for 1 h, after which it was centrifuged at 4°C, 11,000 r/min for 15 min, and the supernatant removed; 1 mL of 70% ethanol (prepared with DEPC water) was added to the solution, centrifuged again, and the ethanol removed. The dsRNA products were then resuspended in 20 μL of ddH_2_O, verified, and kept at –80°C for the following RNA interference experiments. Using the same method, dsGFP was prepared with the primers dsGFP-F and dsGFP-R (Supplementary Table S1), according to the sequence (from 1155 to 1811, 657 bp) of a green fluorescent protein GFP (GenBank accession number: MF169984.1).

### dsRNA Microinjection

Nymphs (2nd instar) were microinjected with dsNlATG1 as the treatment, for which dsGFP served as the control; each group consisted of 60 BPH individuals. The final concentration of purified dsRNA was 5 μg/μL. The microinjection site was located at the coxal cavity of the mesofoot (Liu et al., 2010). Any dead individuals were removed, and the survival was recorded daily. After microinjecting them, the nymphs were placed onto the susceptible rice variety TN1 and reared under the same conditions (26 ± 2°C, humidity of 80 ± 5%, 12-h/12-h light/dark photoperiod).

In addition, a parallel RNA interference treatment was set up to analyze the expression level of the *NlATG1* gene and mitochondrial fission gene *NlFis1* and fusion gene *NlMarf*, with three replicates for each. Each group was sampled every 2 days, and six individuals were randomly selected and frozen in liquid nitrogen and kept at –80°C for subsequent RNA extractions. The RT-qPCR methods, primers, and reaction conditions were the same as those described in subsection 2.5.

### Sample Processing Procedures of TEM

The BPH individuals were dissected under a microscope and first fixed overnight with 2.5% glutaraldehyde, then fixed again with 1% OsO_4_ for 1 h after washing with PBS. Samples were dehydrated by a graded series of ethanol (30, 50, 70, 80, 90, 95, and 100%) and sectioned using a Leica EM UC7 ultratome after being embedded. Sections were stained by uranyl acetate and alkaline lead citrate for 5–10 min and observed using transmission electron microscopy (TEM) under a Hitachi Model H-7650.

### ATP Content Measurement

Approximately 20 mg of a BPH sample treated with dsNlATG1 or dsGFP for 4 days was homogenized with a 150-μL lysis buffer following the instructions of the ATP Assay Kit (Beyotime, Shanghai, China) and centrifuged at 12000 × *g* and 4°C for 5 min. The supernatant (20 μL) was removed for the detection of ATP in a black 96-well plate. The protein concentration was measured simultaneously using the BCA Protein Assay Kit (Beyotime, Shanghai, China) to enable the final calculation of ATP content. Three independent biological replicates and three technical replicates were used to measure ATP in this way.

### Statistical Analysis

The software programs SPSS 20.0 and GraphPad Prism 6.0 were used to analyze the data and draw the figures. ImageJ was used to measure the width and length of mitochondria. All values were presented as the mean ± SD of at least three independent biological replications. Student’s *t*-tests were used to compare means of two groups of samples; one-way ANOVA followed by Tukey’s *post hoc* test was used to compare three or more group means of samples.

## Results

### Full-Length cDNA Cloning and Sequence Characterization of *NlATG1*

The RACE results showed that the *NlATG1* gene had a full-length cDNA of 2502 bp. It was predicted that *NlATG1* contains a 2040-bp ORF (GenBank accession number: MF062504) and encodes a hypothetical protein of 679 amino acids. The NlATG1 protein was estimated to have a molecular weight of 74.3 kDa with an isoelectric point of 8.31. No signal peptide was found for it.

**FIGURE 1:**
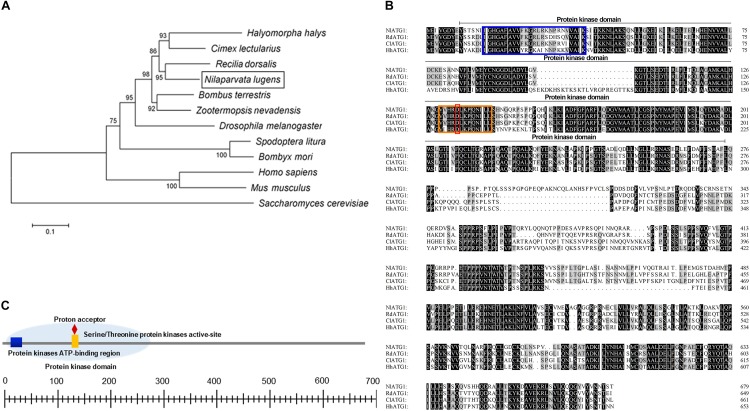
Analysis of the ATG1 protein sequence. **(A)** Phylogenic tree of ATG1 from the brown planthopper (BPH, *Nilaparvata lugens*) and other insect species based on amino acid sequences and using the neighboring-joint method. The tree was constructed in MEGA 5.0, and the phylogeny was tested with the bootstrap method (1000 replications); percentage values >50 are shown on each node. *NlATG1* is shown in a black box. Accession numbers for the sequences of the other species are as follows: *Halyomorpha halys*, XP_014270311.1; *Cimex lectularius*, XP_014254421.1; *Recilia dorsalis*, ATV91623.1; *Bombus terrestris*, XP_003394615.1; *Zootermopsis nevadensis*, XP_021941781.1; *Drosophila melanogaster*, NP_001163433.1; *Spodoptera litura*, AJE75846.1; *Bombyx mori*, NP_001296475.1; *Homo sapiens*, NP_003556.1; *Mus musculus*, NP_038909.3; *Saccharomyces cerevisiae*, KZV11049.1. **(B)** Comparison of four amino acid sequences from *N. lugens*, *H. halys*, *C. lectularius*, and *R. dorsalis.* Amino acids identical in all four proteins are marked in black, with three identical amino acids marked in gray. Blue box, protein kinase ATP-binding region; orange box, serine/threonine protein kinases activity site; red box, proton acceptor. **(C)** Structural analysis of the protein domain from *NlATG1*. The scaled line below indicates the length of the amino acid.

Phylogenetic analysis revealed that ATG1 had homology with different species. The ATG1 protein of BPH was quite similar to *Cimex lectularius* (Hemiptera), *Halyomorpha halys* (Hemiptera), and *Recilia dorsalis* (Homoptera); among them, the most closely related amino acid sequence to *NlATG1* is from *R. dorsalis*, with 95% shared identity (Figure 1A). Multiple amino acid sequence alignment from those four insects showed that all sequences harbor a protein kinase domain, albeit with some differences among them (Figure 1B). The protein kinase domain (PS50011) of *NlATG1* contains a protein kinase ATP-binding region (at positions 15 through 39), a serine/threonine protein kinases activity site (at positions 130 through 142), and a proton acceptor (at position 134) (Figure 1C). Based on analysis of its structure, the physiological function of ATG1 protein is predicted to be similar to its homologous counterparts in other insect species.

### Expression Pattern of *NlATG1* in BPH

**FIGURE 2:**
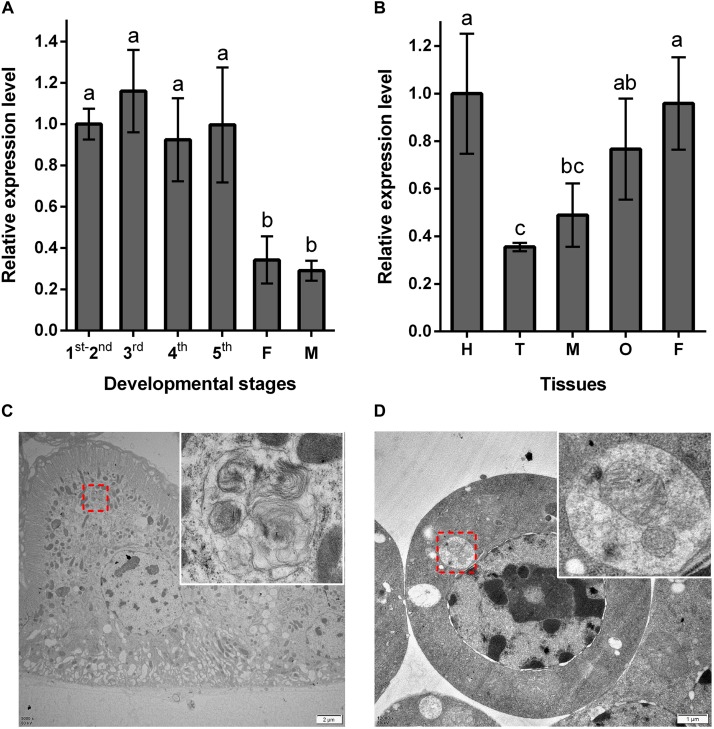
Expression patterns of *NlATG1* in BPH. **(A)** Expression patterns of *NlATG1* at different developmental stages. The mRNA level was normalized relative to the *RPS11* levels, and the reference was the mRNA level of 1st–2nd instar nymphs. 1st–5th: BPH nymphs from 1st instar to 5th instar, F: female adults, M: male adults. **(B)** Relative expression of *NlATG1* in different insect tissues. H: head, T: thorax, M: midgut, O: ovary, and F: fat body. The reference used was the mRNA level of the head. Relative gene expression was compared by Tukey’s test, for which a, b, and c on the bars indicate significant differences among each sample (*p* < 0.05). **(C,D)** Autophagy occurred in midgut cells and fat body cells examined under a transmission electron microscope. The red dotted box denotes the autophagic vacuoles that are enlarged in the top right-hand corner. Scale bars: 2 μm in **(C)**; 1 μm in **(D)**.

Using RT-qPCR, the expression pattern of *NlATG1* was investigated in BPHs raised on susceptible TN1 rice. These results demonstrated that *NlATG1* was expressed in different developmental stages, but at higher levels in nymphs and lower levels in both male and female adults (Figure 2A). Generally, the expression level in adults began to decrease after eclosion, reaching its lowest level at day 5, after which it increased slightly over the following days. Additionally, the transcription level of *NlATG1* was not correlated with either macropterous or brachypterous wing forms, also showing no correlation with females or males in the adult stage (Supplementary Figure S1). Concerning the 5th instar nymphs, *NlATG1* had different expression levels in them in different tissues, being higher in the head, midgut, ovary, and fat body but lowest in the thorax (Figure 2B). The TEM revealed that autophagy did occur in certain tissues, such as the midgut and fat body, with some mitochondria found in autophagosomes undergoing mitophagy (Figures 2C,D).

### RNAi of *NlATG1* Reduced the Survival of BPH Juveniles and Adults

**FIGURE 3:**
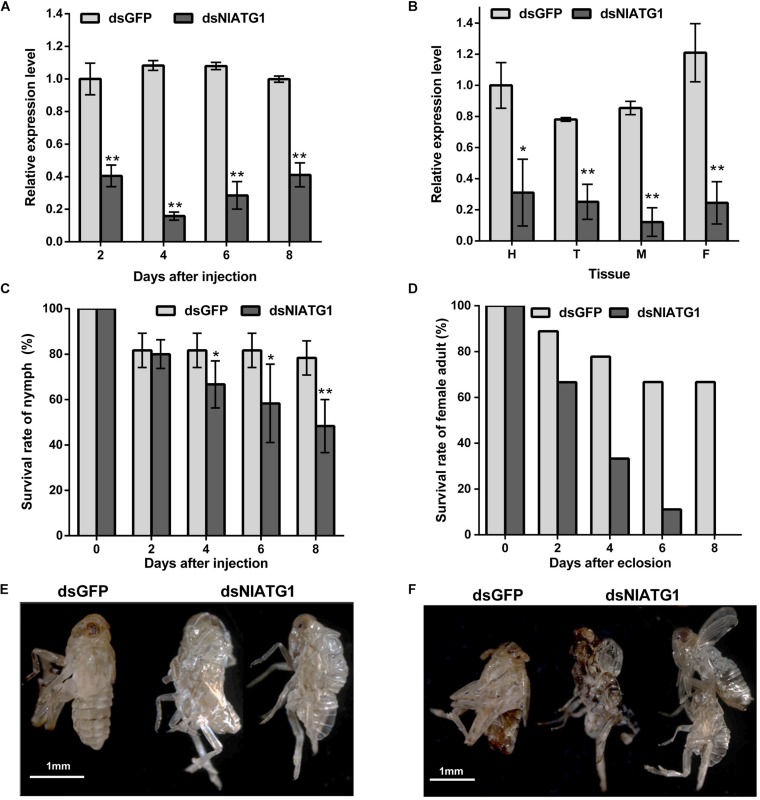
Effects of *NlATG1* gene RNA interference on BPH. **(A,B)** Effect on the mRNA expression level of *NlATG1* microinjected with dsRNA at different treatment times **(A)** and in different tissues at day 4 **(B)**. The reference was the mRNA level of BPH treated with dsGFP at day 2 **(A)** and head **(B)**. **(C,D)** Effect of *NlATG1* RNAi on the survival rate of BPH nymph **(C)** and female adult **(D)**. All values are presented as the means ± SD of three independent replicates (*p < 0.05, **p < 0.01). **(E,F)** Some BPH died of failure in molting and eclosion; scale bars: 1 mm.

The qPCR results showed that the expression level of *NlATG1* was significantly knocked down after RNA interference, decreasing to 40.5, 14.6, 26.4, and 41.2 of the dsGFP control group at days 2, 4, 6, and 8, respectively (Figure 3A). Hence, the efficacy of RNA interference peaked at day 4 but then diminished over time. The expression level of *NlATG1* in different tissues was also significantly knocked down at day 4, decreasing to 31.1, 32.2, 14.3, and 20.3% of the control group in the head, thorax, midgut, and fat body, respectively (Figure 3B).

The injection of dsNlATG1 caused a significant decrease in BPH survival from day 4. At day 8, the survival of the treated group had decreased to 48.3%, whereas the control group maintained a higher survival of 78.3% (Figure 3C). It is worth noting that RNAi of dsNlATG1 considerably lowered the survival of female adults, such that all dsNlATG1-treated females had died within 8 days after eclosion, while the control group’s survival was higher at 66.7% (Figure 3D). In addition, some BPHs died of molting failure (Figures 3E,F).

### RNAi of *NlATG1* Inhibited Autophagy in Epithelial Cells of the Midgut in BPH

**FIGURE 4:**
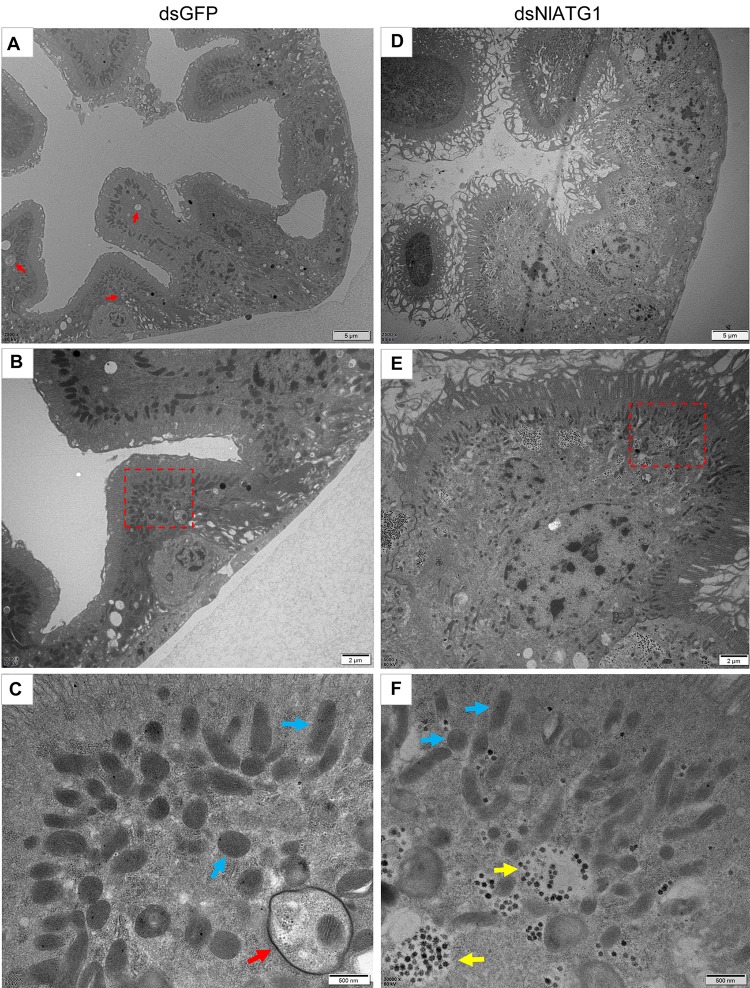
Transmission electron microscopy graphs of epithelial cells in the midgut of BPH **(A–C)** treated with dsNlATG1 and **(D–F)** treated with dsGFP, at day 4. Red arrows denote autophagic vacuoles or compartments, blue arrows denote mitochondria, and yellow arrows denote glycogen. **(C)** Enlargement of the part in a red dotted box in **(B)**. **(F)** Enlargement of the part in a red dotted box in **(E)**. Scale bars: **(A,D)**, 5 μm; **(B,E)** 2 μm; **(C,F)** 500 nm. At least three biological replicates were performed per treatment.

The TEM results showed that in normally feeding BPHs, the epithelial cells of their midgut underwent basic autophagy with several autophagic vacuoles or autophagic compartments, including mitochondrial autophagy (mitophagy) (Figures 4A–C). In stark contrast, almost no vacuoles were observed in those cells interfered with by dsNlATG1 (Figures 4D–F). Further, RNA interference with dsNlATG1 also affected the metabolism of glycogen, such that glycogen accumulated in the midgut cells (Figure 4F).

**FIGURE 5:**
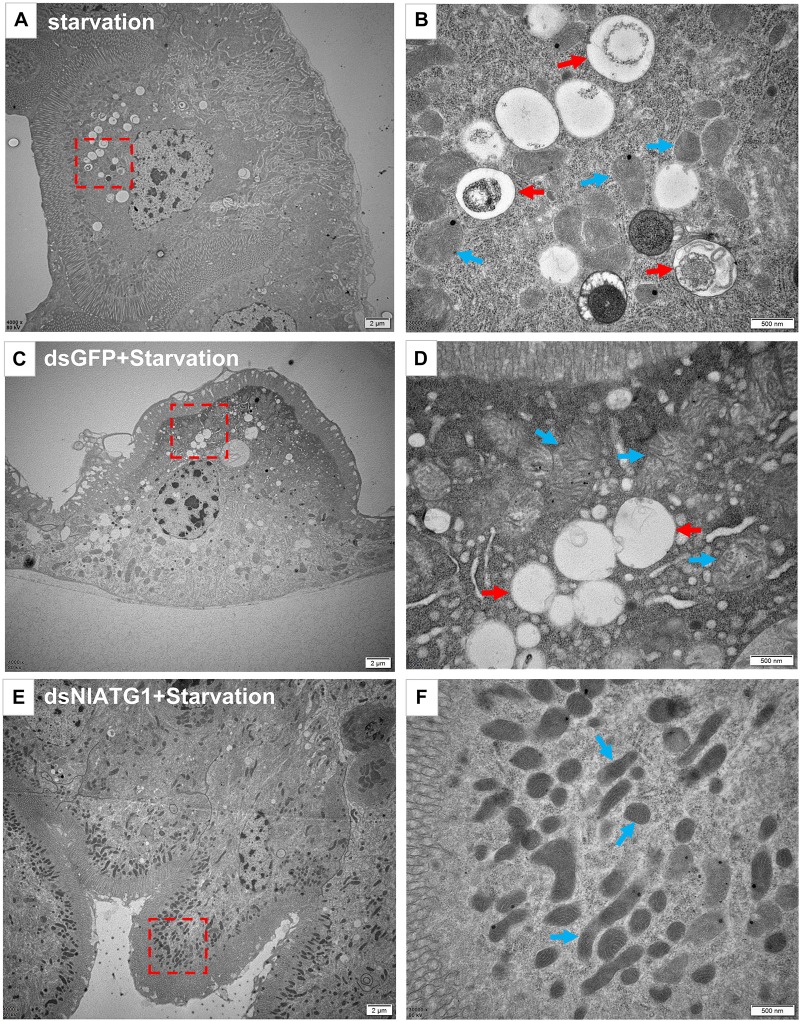
Transmission electron microscopy graphs of midgut cells. **(A,B)** The control group of the starvation treatment at 48 h; **(C,D)** starvation treatment at 48 h after injecting dsGFP for 4 days. **(E,F)** starvation treatment for 48 h after injecting dsNlATG1 for 4 days. Red arrows denote autophagic vacuoles or compartments, while blue arrows denote the mitochondria. **(B**,**D)** and **(F)** represent enlargements of the parts indicated by red dotted boxes in **(A**,**C)** and **(E)**, respectively. Scale bars: **(A,C,E)** 2 μm; **(B,D,F)** 500 nm. At least three biological replicates were used per treatment.

To further validate the effect of RNA interference of *NlATG1* upon autophagy, the BPH was first injected with dsRNA and then left to starve. TEM examinations showed that autophagy was evidently induced in the starving midgut cells of dsGFP-treated BPH individuals (Figures 5C,D), with similar autophagy vacuoles as in starving non-treated individuals (Figures 5A,B). By contrast, autophagy was clearly inhibited in the dsNlATG1-treated group, with almost no vacuoles visibly formed in the midgut cells even under starvation conditions (Figures 5E,F).

### Effects of *NlATG1* RNAi on Mitochondrial Morphosis

**FIGURE 6:**
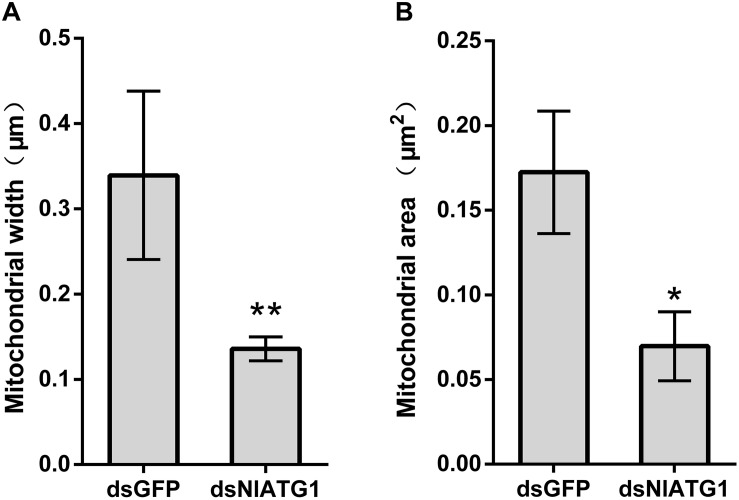
Mitochondrial width and mitochondrial area when treated with dsRNA. **(A)** mitochondrial width; **(B)** mitochondrial area. All values are presented as the mean ± SD of three independent biological replicates (*p < 0.05, **p < 0.01). (Approximately 100 mitochondria were measured per replicate).

**FIGURE 7:**
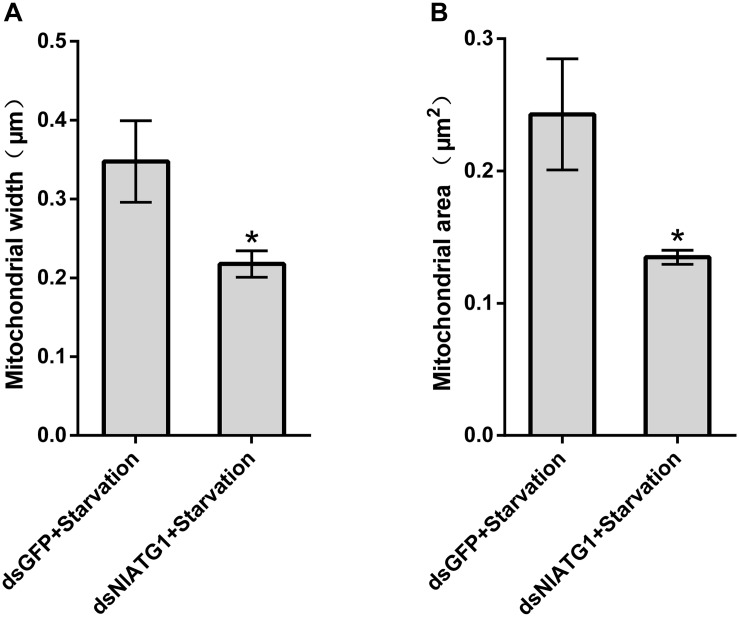
Mitochondrial width and mitochondrial area when treated with dsRNA and starvation. **(A)** mitochondrial width; **(B)** mitochondrial area. All values are presented as the means ± SD of three independent biological replicates (*p < 0.05). (Approximately 100 mitochondria were measured per replicate).

TEM graphs showed that the mitochondria in cells treated with dsGFP were generally long or short and rod-like, sectioned into different shapes (e.g., spherical or oval and tubular; Figure 4C). Compared with the dsGFP group, the shape of mitochondria treated with dsNlATG1 did not change much, but they did become narrower (Figure 4F). The width (diameter) of mitochondria in the dsNlATG1-treated groups (0.14 μm) was approximately 41% that of the dsGFP control group (0.34 μm) (Figure 6A). Accordingly, the section areas of the mitochondria in the dsNlATG1-treated groups were also reduced (Figure 6B). Moreover, mitochondrial width and area also decreased in the BPH group first treated with dsNlATG1 and then left to starve (Figures 7A,B). Collectively, these results indicated the mitochondria treated with dsNlATG1 became narrower or smaller than the control.

### RNAi of *NlATG1* Upregulated the Mitochondrial Fission Related Gene *NlFis1*

**FIGURE 8:**
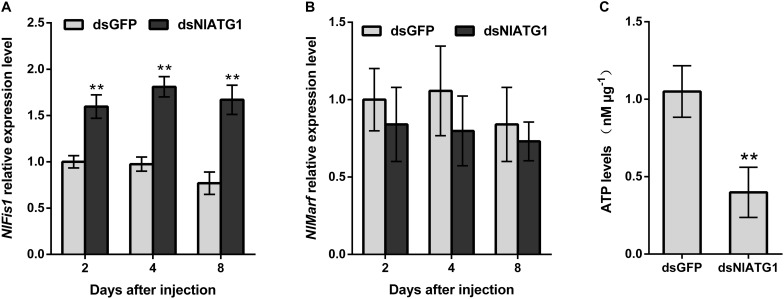
Relative expression level of the mitochondrial fission gene *NlFis1* and fusion gene *NlMarf*, and the ATP content of BPH. **(A)** Expression level of *NlFis1* gene. **(B)** Expression level of *NlMarf* gene. **(C)** The ATP content. BPH individuals were treated with dsRNA for 4 days. All values are presented as the mean ± SD of three independent biological replicates (**p < 0.01).

To better understand how the narrower mitochondria formed, the expression levels of mitochondrial fission gene *NlFis1* and fusion gene *NlMarf* were both analyzed. According to the RT-qPCR, the mRNA level of *NlFis1* increased significantly in the dsNlATG1-treated groups, increasing by 117.2% at day 8 when compared with that of the control (Figure 8A). However, the mRNA transcription level of *NlMarf* showed no significant change (Figure 8B). Therefore, the narrower or smaller mitochondria should have arisen from mitochondrial fission, regulated by the *NlFis1* gene.

### RNAi of *NlATG1* Decreased the ATP Content

Because ATP is mainly synthesized in the mitochondria, ATP content can reliably reflect the functioning of this organelle. To examine whether the RNAi of *NlATG1* affected mitochondrial function in ATP synthesis, the ATP content was determined. This showed that injection of dsNlATG1 significantly decreased the ATP levels, in that the ATP content in the treated groups (0.4 nM μg^–1^) was approximately 36% that of the dsGFP control group (1.1 nM μg^–1^). Hence, RNAi of *NlATG1* altered mitochondrial functioning in the BPH’s synthesis of ATP (Figure 8C).

## Discussion

In this study, we identified an autophagy-related gene, *NlATG1*, and demonstrated that it is essential for autophagy in an insect pest of rice, the brown planthopper (BPH). We found that RNA interference targeting the *ATG1* gene significantly inhibited the autophagy and decreased the survival of BPH, especially reducing the survival of its female adults. The number of females is an important factor affecting the insect’s population size and growth, so we suggest that it offers a promising approach to controlling BPH by inhibiting *ATG1* gene expression and autophagy. Furthermore, RNA interference of *NlATG1* upregulated the *NlFis1* gene involved in mitochondrial fission, leading to reductions in mitochondrial width and area. Knockdown of *NlATG1* also decreased the ATP content and accumulation of glycogen. As far as we know, this is the first time that the *ATG1* gene, as well as autophagy, has been systematically studied in the incompletely metamorphosed insects such as BPH. Previous studies on insect autophagy were mostly carried out at the cellular level of completely metamorphosed insects, focusing mainly on the regulatory mechanism of autophagy, but they rarely explored its effect on the survival of individuals (Lee et al., 2002; Scott et al., 2007; Casati et al., 2012; Liu et al., 2013; Franzetti et al., 2016; Zhan et al., 2018). Therefore, our work here identified a new potential target, *NlATG1*, for controlling BPH and sheds fresh light on the autophagy in an incompletely metamorphosed insect, which is of great significance for developing novel RNAi-based pest control strategies.

Compared with chemical pesticides, employing an RNAi-based strategy is an environmentally friendlier way to control insect pests, since it can be designed specifically to control the target insect pest without impacting beneficial insects or other animals such as pollinators or pest parasitoids (Scott et al., 2013; Yu et al., 2016; Cooper et al., 2019; Liu et al., 2019). For example, species-specific dsRNAs for *Snf7* or *V-ATPaseE* killed the targeted insects without affecting non-targeted species (Whyard et al., 2009; Bachman et al., 2013; Liu et al., 2019). In our study, phylogenetic analysis showed that the NlATG1 protein of BPH closely resembles the ATG1 protein (ATV91623.1) of *R. dorsalis*. By aligning the mRNA sequence of NlATG1 (MF062504) from BPH and the mRNA sequence of ATG1/ULK1 (MF038047.1) from *R. dorsalis*, two matches were submitted with respective identities of 74% (8 gaps) and 68% (28 gaps). According to Scott et al. (2013), single mismatches between the target gene and dsRNA generally impair the RNAi effect; so we think that the sequencing of more and more insect genomes will greatly enhance the possibility of designing suitable dsRNA specifically targeting the *NlATG1* gene of BPH without causing off-target effects in the field. Current dsRNA treatment modes, such as feeding, microinjections, spraying, and GMO-based strategies, are popular for insects (Araujo et al., 2006; Turner et al., 2006; Baum et al., 2007; Mao et al., 2007; Wang et al., 2011). However, it remains a formidable task to deliver dsRNA into a piercing–sucking insect in the agricultural industry, and BPH is no exception. For example, piercing–sucking insects feed on phloem sap while ingesting very little plant tissue, so foliar application of dsRNA by spraying is not likely to trigger an effective RNAi response in them. Microinjection is an effective strategy for delivering dsRNA into many orders of insects, including BPH (Wang et al., 2017; Zhu et al., 2017; Waris et al., 2018; Ge et al., 2019), but this approach is not a feasible strategy for achieving widespread crop protection in the field (Huvenne and Smagghe, 2010; Scott et al., 2013; Liu et al., 2019). Recently, Li et al. (2015) suggested that soaking the root system in dsRNA could be used as a dsRNA-delivery strategy during crop irrigation, which is also suitable for BPH control. Therefore, the next step in our work will focus on how to practically couple the *NlATG1* gene with an RNA-based pest-control strategy.

We found that autophagy occurred at all developmental stages of BPH, especially during its nymphal stages. RNAi of *NlATG1* resulted in mortality of both nymphs and adults but decreased the survival of female adults the most (Figure 3C). It is unclear whether treatment with dsNlATG1 resulted in greater mortality of females than males as nymphs, for it is difficult to distinguish females from males when they are immature. According to our study’s RNAi results, autophagy plays multiple roles, not only in molting but also in maintaining the homeostasis of mitochondria and regulating the metabolism of glycogen. Yet it is still uncertain which of these, upon interference, is the main reason for BPH mortality. Therefore, why the RNAi of *NlATG1* induced more female adults to die than male counterparts requires further investigation.

The finding in this study that *NlATG1* was expressed in different organs/tissues further indicated that this gene should play multiple roles in BPH. Similarly, in a previous study, *BmATG1* was found to be expressed in different tissues of *Bombyx mori*, especially in its larval midgut during the larval-to-pupal transformation (Casati et al., 2012). Earlier, it was shown that *D. melanogaster* had great sensitivity to starvation and possible failure in pupation stages under conditions of insufficient *DmATG1* (Scott et al., 2007; Hou et al., 2008). However, those studies on the autophagy of complete metamorphosis insects mainly focused on pupa or larval–adult transformation, leaving much information unclear in incomplete metamorphosis insects. Therefore, our findings here for BPH provided new information on autophagy in the midgut of an incompletely metamorphosing insect. Mitochondria are abundant in the epithelial cells of the midgut, where they supply the large amounts of ATP required for transporting nutrients from the intestinal lumen to the cytoplasm of the epithelial cell (Manjunatha et al., 2018). From this point of view, the epithelial cell of the midgut offers a good model for analyzing autophagy, and, in particular, mitochondrial autophagy (mitophagy) (Lee et al., 2002; Lőrincz et al., 2017).

Mitochondria maintain their homeostasis through the dynamic equilibrium of fission/fusion (Arun et al., 2016). During this process, the mitochondria periodically exchange proteins, mtDNA, and lipids via rapid and alternating fusion and fission (Twig et al., 2008b). Nevertheless, mitochondrial fission/fusion defects will limit mitochondrial movement, resulting in reduced energy production and oxidation (Knott and Bossy-Wetzel, 2008). Proteins or mtDNA in mitochondria are easily damaged, so mitochondria must carry out fusion and fission events to facilitate the adequate exchange of vital organelle contents (Twig et al., 2008a). In our study, *NlFis1* expression was detected that increased upon *NlATG1* interference, but the fusion-related gene *NlMarf* did not vary significantly, thus indicating that the dynamic balance of fission and fusion was lost, which could explain the mitochondrial accumulation we found. In cases where their fusion is blocked, the mitochondria become dysfunctional and consume cytoplasmic ATP to maintain their membrane potential (Knott and Bossy-Wetzel, 2008; Gomes et al., 2011). To test whether the fission of mitochondria resulted in more mitochondria, we determined the copy number of a *cox2* gene in mtDNA using qPCR, normalized to the *Ndufs7* gene in BPH’s genomic DNA. However, the relative copy number of the *cox2* gene failed to change significantly after the RNAi of *NlATG1* (Supplementary Figure S2). Just over 10 years ago, it was suggested that fission events may be indispensable for autophagy (Twig et al., 2008a). Fission is more likely to separate dysfunctional parts, including the mtDNA, from the mitochondrial network (Twig et al., 2008a). As is known, one or more mtDNAs usually exist in a mitochondrion (Rand, 2001), so during the fission process, random separation of mtDNA leads to the separation of defective mtDNAs (Twig et al., 2008b). We presumed that during the inhibition of autophagy in BPH through the RNAi of *NlATG1*, the fission of its mitochondria was upregulated, yet their fusion went unaffected, so more damaged mitochondria accumulated and thereby affected the functioning of this insect’s mitochondria. However, the mechanism of how RNAi of *NlATG1* affected the fission of mitochondria needs to be further explored.

## Conclusion

In conclusion, our work here identified an autophagy-related gene of BPH, *NlATG1*, and revealed both its developmental and spatiotemporal expression profiles. RNA interference and TEM results confirmed that the *NlATG1* gene plays a key role in the autophagy of this insect. RNA interference of the *NlATG1* gene caused elevated mortality, suggesting that it is a promising target for management of this pest.

## Data Availability Statement

The datasets generated in this study have been deposited in the GenBank database (accession numbers MF169984.1 and MF062504).

## Author Contributions

PH and XY designed the experiments. FY, CY, YF, and KP conducted the experiments. PH, XY, and FY wrote and edited the manuscript.

## Conflict of Interest

The authors declare that the research was conducted in the absence of any commercial or financial relationships that could be construed as a potential conflict of interest.

## Footnotes

1 https://www.ncbi.nlm.nih.gov/orffinder/

2 http://web.expasy.org/protparam/

3 http://www.cbs.dtu.dk/services/SignalP/

4 http://prosite.expasy.org/
