# Supplementary material for: NlATG1 Gene Participates in Regulating Autophagy and Fission of Mitochondria in the Brown Planthopper, Nilaparvata lugens
Source: Front Physiol. 2020 Jan 31;10:1622. doi: 10.3389/fphys.2019.01622 (PMC7004972; doi:10.3389/fphys.2019.01622)
Supplement: Supplementary file 1 [file Data_Sheet_1.docx]

Supplementary Material

## Supplementary Figures

**
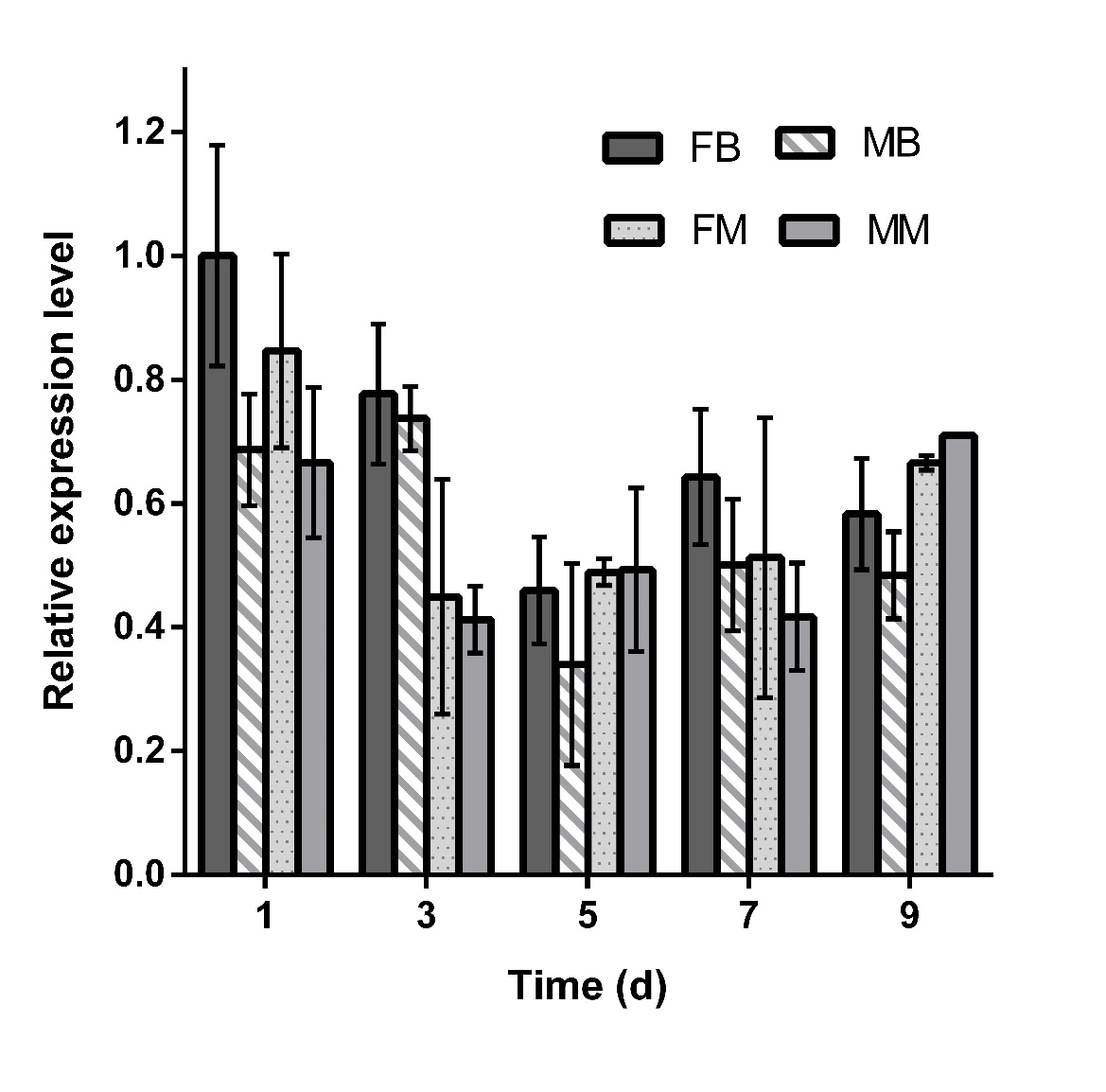
**

**Supplementary Figure 1.** Expression patterns of *NlATG1* in adults at different days after eclosion. The mRNA level was normalized relative to the *RPS11* levels, and the reference was the mRNA level of BPH 1 day after eclosion. FB: female brochypterous; MB: male brochypterous; FM: female macropterous; MM: male macropterous.


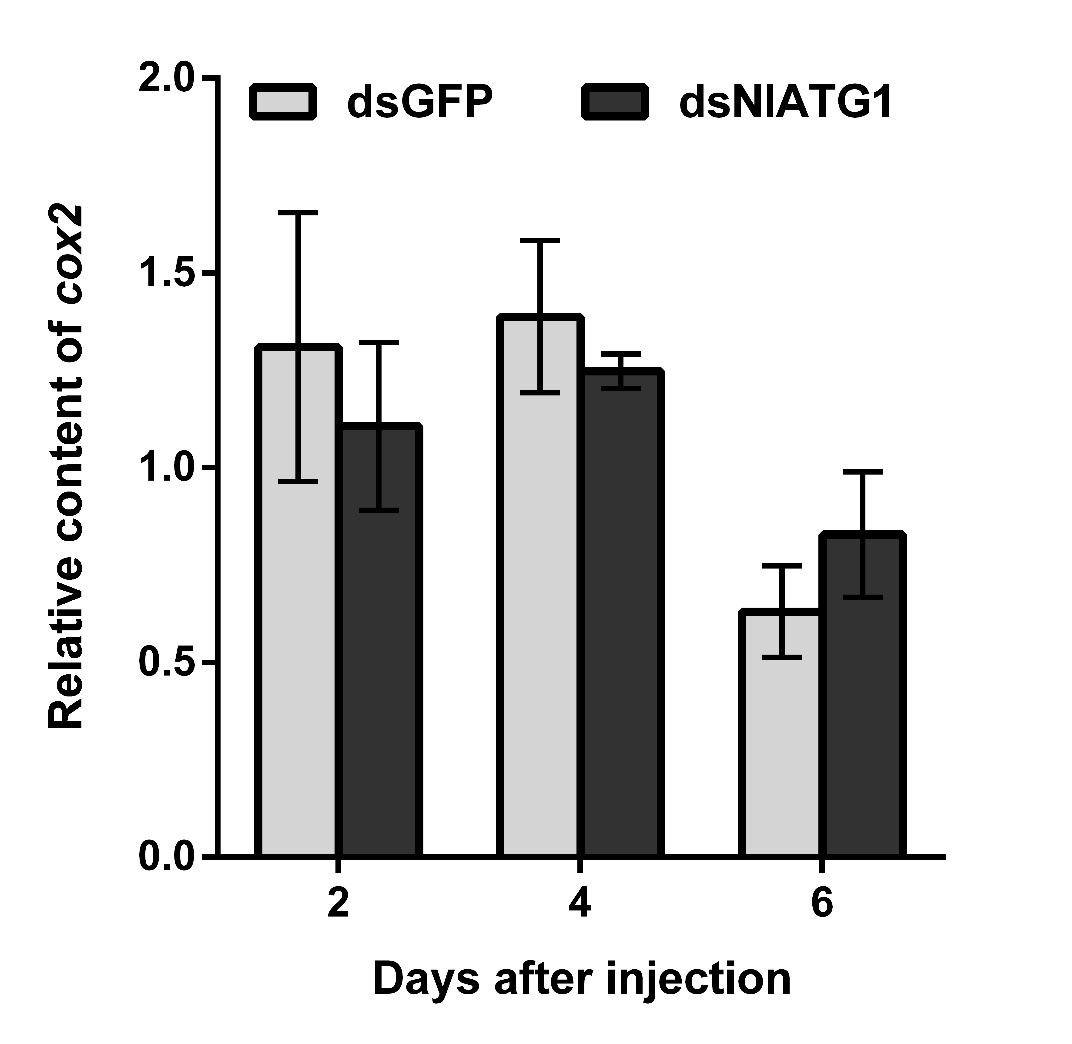


**Supplementary Figure 2.** Mitochondrial DNA (mtDNA) content after dsNlATG1 injection. MtDNA was quantified by qPCR, normalized relative to the nuclear gene as an internal reference. The mitochondrial-encoded gene was *cox2*, and the nuclear-encoded gene was *Ndufs7*. The relative copy number of genes was analyzed by the 2^–ΔΔCt^ method. The primers for mitochondrial DNA are given in Table S1. All values are presented as the means ± SD of three independent replicates.

## Supplementary table

| **Name** | **Sequence （5'→ 3'）** |
| --- | --- |
| *Primers for cDNA cloning* |  |
| NlATG1-F | ATGAGAATGTCGTGGCGTTG |
| NlATG1-R | ACCGCCAGCACAAAGTTCAG |
| NlATG1-3′RACE outer | GTTATGGCTGCTACGCTGTGTGG |
| NlATG1-3′RACE inner | TCACAGTTTCCCAGTTTGCCTC |
| NlATG1-5′RACE outer | TGAGAAAGGGCGAACTGACCACT |
| NlATG1-5′RACE inner | TCATCGGGAGGCGTTTGGTTTT |
| NlATG1-FL-F | CGGAGACAGAAGGCAGATTG |
| NlATG1-FL-R | TTCCAATACCCCAACTAATG |
| *Primers for real-time qPCR* |  |
| NlATG1-qF | GACGCCTTCTTTTCGCACGC |
| NlATG1-qR | GGAAGACAACTGGGTGGGGG |
| NlRPS11- qF | CCGATCGTGTGGCGTTGAAGGG |
| NlRPS11- qR | ATGGCCGACATTCTTCCAGGTCC |
| cox2- qF | CACAGATTTCGGAGCAT |
| cox2 qR | CTTCCTTACCCAACTAC |
| Ndufs7- qF | ATTCTATCGCATCCTCT |
| Ndufs7- qR | CTCGCTGGGTTATTTCA |
| NlFis1- qF | GCATCGGAGACAGGACA |
| NlFis1 qR | CCTTTGCGTACATCAGC |
| NlMarf- qF | ATGGCAAAGCTACTACCC |
| NlMarf- qR | CTCCACGAGAACCTGAACT |
| *dsRNA synthesis* |  |
| dsNlATG1-F | GGATCCTAATACGACTCACTATAGGGCTGGCGGTGAGCGAGTGCGTGA |
| dsNlATG1-R | GGATCCTAATACGACTCACTATAGGGTCTTCCAATACCCCAACTAATG |
| dsGFP-F | GGATCCTAATACGACTCACTATAGGGAAGGGCGAGGAGCTGTTCACCG |
| dsGFP-R | GGATCCTAATACGACTCACTATAGGGCAGCAGGACCATGTGATCGCGC |

**Supplementary Table 1.** PCR primers used in the study
